# Machine learning reveals an interleukin-33-associated immuno-metabolic axis defining recovery endotypes in severe combat trauma

**DOI:** 10.3389/fimmu.2026.1906285

**Published:** 2026-08-10

**Authors:** Vladyslav Bardash, Oleh Samchuk, Igor Skrypnyk, Eugen Sklyarov

**Affiliations:** 1 Center for Nephrology and Dialysis, St. Panteleimon Hospital, First Lviv Medical Union, Lviv, Ukraine; 2 General Directorate, First Territorial Medical Union of Lviv, Lviv, Ukraine; 3 Department of Internal Medicine No. 1, Poltava State Medical University, Poltava, Ukraine; 4 Department of Therapy No. 1, Medical Diagnostics, Hematology, and Transfusiology, Faculty of Postgraduate Education, Danylo Halytsky Lviv National Medical University, Lviv, Ukraine

**Keywords:** combat trauma, endothelial glycocalyx, interleukin-33, machine learning, molecular endotypes, precision medicine

## Abstract

Severe combat trauma is associated with long-lasting systemic changes, yet standard anatomical injury scales may not adequately capture biological differences in patient recovery. To explore this variability, we analyzed the systemic molecular profiles of 53 combat trauma survivors during the rehabilitation phase and compared them to those of 46 uninjured active-duty military controls. Using a multidimensional biomarker panel (IL-33, ST2, TGF-β1, CTGF, galectin-3, heparan sulfate, NT-proBNP, and annexin A5) alongside routine clinical parameters, we applied unsupervised clustering and Random Forest machine learning to identify distinct patient endotypes. Our results showed that patients grouped into distinct molecular phenotypes, independent of their initial structural injury patterns. While localized structural injuries and associated infections (e.g., limb amputations) significantly influenced pro-fibrotic and pro-coagulant pathways, the overarching systemic features defining the machine-learning endotypes closely linked to an immuno-metabolic axis. This axis is characterized by elevated IL-33, endothelial glycocalyx shedding, and markers of metabolic, hepatic, and hematological strain (hemoglobin, white blood cells, AST, ALT, and total protein) rather than classical fibrotic markers. Notably, while circulating IL-33 levels varied significantly among patients, its soluble receptor, ST2, remained stable. These findings suggest that high-risk trauma patients may experience ongoing, ligand-driven systemic exhaustion. Integrating molecular endotyping into military medicine could improve clinical monitoring and guide targeted rehabilitation strategies.

## 1 Introduction

Modern combat trauma, characterized by severe blast-induced and penetrating injuries, triggers a protracted state of systemic dysregulation that persists long after the initial surgical intervention (1, 2). This response is driven by a complex molecular cascade, including endothelial glycocalyx degradation (heparan sulfate) (3, 4), the release of damage-associated molecular patterns (DAMPs) such as IL-33 (Interleukin-33) (5–7), and a sustained pro-fibrotic shift mediated by connective tissue growth factor (CTGF) and transforming growth factor-beta 1 (TGF-β1) (8, 9). While these biomarkers signal significant physiological instability, traditional anatomical injury scales often fail to capture the underlying biological diversity of the host response (10). By utilizing uninjured military personnel as a baseline control, we aim to isolate the true molecular signature of combat trauma, distinguishing injury-specific dysregulation from the inherent physiological stress of military service (11).

Despite sharing similar injury patterns, military trauma patients exhibit heterogeneous molecular trajectories in the rehabilitation phase of recovery (12). This variability suggests the existence of distinct **“**endotypes**”** — subgroups defined by unique pathobiological mechanisms rather than clinical labels alone (13). To resolve these multidimensional patterns, we employed an integrative computational framework combining unsupervised hierarchical clustering with supervised Random Forest algorithms (14, 15). This approach enables the discovery of underlying molecular patterns and the identification of primary phenotypic drivers, such as tissue-damage and metabolic axes. Consequently, our study seeks to clarify the systemic molecular landscape of military trauma, providing a scalable model for precision monitoring and biomarker discovery in heterogeneous survivor populations.

While the acute systemic response to combat trauma has been extensively studied, a significant knowledge gap remains regarding the long-term biological trajectories of survivors during the rehabilitation phase. Specifically, the interplay between persistent systemic inflammation, endothelial damage, and tissue remodeling months or years after injury requires deeper investigation. To address this, we evaluated a comprehensive, interconnected panel of biomarkers designed to capture core pathobiological axes: damage-associated molecular patterns (IL-33/ST2), endothelial glycocalyx degradation (heparan sulfate proteoglycan and annexin V), and extracellular matrix remodeling (TGF-β1, CTGF, and galectin-3). A sustained pro-fibrotic shift is a critical clinical challenge in major trauma survivors, as it drives chronic scar formation, tissue stiffness, and functional impairment in healing wounds and residual limbs. By integrating these markers with routine clinical and microbiological data — including detailed pathogen profiling of persistent wound infections—we aim to resolve the biological heterogeneity of trauma survivors and identify stable recovery endotypes beyond traditional anatomical classifications, such as limb amputation status.

The primary objective of this study was to characterize the systemic molecular landscape of military trauma in the rehabilitation phase and to determine whether these alterations converge into stable biological endotypes. By utilizing a cohort of uninjured military personnel as a rigorous baseline, we evaluated a comprehensive panel of biomarkers reflecting endothelial integrity, hemodynamic stress, and tissue remodeling. We hypothesized that combat-related trauma is associated with a persistent state of systemic dysregulation that remains stable over time, and that unsupervised machine learning can resolve this heterogeneity into distinct molecular phenotypes. Specifically, we aimed to identify the primary biological drivers — such as those mediated by tissue-damage markers or metabolic axes — that define these endotypes. Our findings provide a framework for the precision monitoring of trauma survivors and offer new insights into the hierarchical nature of the host response to severe structural injury.

## 2 Materials and methods

### 2.1 Study design and ethical approval

This was a single-center, cross-sectional, observational study designed to evaluate the systemic molecular and immuno-metabolic profiles of military personnel during the rehabilitation phase of recovery following severe combat-related trauma. The study protocol was conducted in strict adherence to the ethical principles outlined in the Declaration of Helsinki and its later amendments.

The research protocol, including all clinical data collection and blood sampling procedures, was reviewed and officially approved by the Institutional Ethics Committee of Danylo Halytsky Lviv National Medical University (Protocol Number: No. 10, Date of Approval: September 10, 2023). Prior to enrollment and any study-related interventions, written informed consent was obtained directly from all participating individuals.

### 2.2 Study population and setting

The study cohort comprised 53 male military personnel (aged 25 to 60 years) who sustained severe combat-related mine-blast injuries of varying severity. A critical inclusion criterion was that all patients were in the late rehabilitation phase, with the most recent injury occurring at least 3 months prior to blood sampling. At the time of enrollment, all patients were clinically stable, hemodynamically compensated, and free from acute life-threatening systemic conditions (e.g., acute sepsis or shock). They were admitted primarily for late-stage reconstructive procedures, such as surgical preparation of residual limbs for prosthetics, stoma closure, or reconstructive plastic surgery. While no acute systemic decompensation was observed during the study period, a subset of patients presented with persistent localized wound or stump infections. To ensure baseline physiological comparability and minimize confounding bias, individuals with a known history of severe chronic medical conditions prior to injury (e.g., diabetes mellitus, severe arterial hypertension, or autoimmune disorders) were strictly excluded from both the trauma and control cohorts.

To establish an appropriate physiological baseline, we enrolled a control group consisting of 46 uninjured active-duty male military volunteers (aged 25 to 60 years) serving in rear-echelon support roles, screened via outpatient medical records to ensure the absence of active chronic diseases. This control group allowed for ribonucleic and molecular isolation of trauma-specific signatures from the standard physiological strain inherent to military service. For all participants, demographic characteristics, age, and available clinical and injury profiles — including specific anatomical deficits (e.g., traumatic or surgical amputations) — were systematically recorded prior to biomaterial collection.

Infection status was systematically evaluated using a comprehensive clinical and laboratory algorithm. Persistent localized wound or stump infections were diagnosed based on a positive microbiological isolation (wound or residual limb stump culture) combined with at least two standardized systemic inflammatory criteria: C-reactive protein (CRP) ≥ 30 mg/L, procalcitonin > 0.5ng/mL, or leukocyte count > 10×10^9^. Detailed pathogen profiling, including exact bacterial strain identification and antimicrobial resistance patterns, was performed for cases with confirmed microbiological isolation.

### 2.3 Blood sampling and biomarker quantification

Peripheral venous blood samples were collected from all participants (both trauma patients and controls) under standardized conditions in the morning after an overnight fast (8–12 hours) to minimize the impact of diurnal variations on biomarker concentrations. To obtain serum, whole blood was drawn into sterile serum separator tubes (SST) without anticoagulant and allowed to clot at room temperature for 30 minutes. The samples were subsequently centrifuged at 1,000 × *g* for 15 minutes at 4 °C to separate the serum. The resulting serum aliquots were immediately frozen and stored at −80 °C until biochemical analysis to strictly avoid multiple freeze-thaw cycles.

This specific biomarker panel was predefined to comprehensively evaluate three core pathophysiological axes of late-stage trauma recovery: alarmin release, extracellular matrix remodeling, and endothelial degradation. No other exploratory biomarkers were measured and excluded from this analysis. Notably, heparan sulfate was specifically selected to assess the endothelial glycocalyx rather than syndecan-1. While syndecan-1 is a well-established marker of acute trauma-induced coagulopathy and acute hemorrhagic shock, heparan sulfate represents the most abundant glycosaminoglycan of the glycocalyx and its enzymatic shedding is deeply implicated in persistent, low-grade systemic inflammation and chronic tissue remodeling, making it highly relevant for the rehabilitation phase.

The quantification of circulating systemic biomarkers was performed using commercially available enzyme-linked immunosorbent assay (ELISA) kits in strict accordance with the manufacturers’ instructions. The target analytes and corresponding assay kits were:

- IL-33: Human IL-33 ELISA Kit (FineTest, Cat. No. EH0198).
- TGF-β1: Human TGF-β1 ELISA Kit (FineTest, Cat. No. EH0287).
- CTGF: Human Connective Tissue Growth Factor ELISA Kit (FineTest, Cat. No. EH0702).
- Galectin-3: Human GAL3 ELISA Kit (FineTest, Cat. No. EH0145).
- Heparan sulfate proteoglycan: Human HSPG ELISA Kit (FineTest, Cat. No. EH3244).
- NT-proBNP: Human NT-proBNP ELISA Kit (FineTest, Cat. No. EH0350).
- Annexin A5: Human ANXA5 ELISA Kit (FineTest, Cat. No. EH0423).

To rigorously validate the stability of the soluble IL-33 receptor and ensure the robustness of our immuno-metabolic findings, circulating ST2 (IL1RL1) levels were cross-validated using two independent, parallel assay platforms: the Human IL1RL1 ELISA Kit (FineTest, Cat. No. EH0188) and the high-performance Human ST2 SimpleStep ELISA Kit (Abcam, Cat. No. ab254505). Statistical cross-platform validation demonstrated a significant positive correlation between the measurements obtained from these two distinct ST2/IL1RL1 assay platforms (Spearman’s *ρ* = 0.377, p < 0.001).

All ELISA procedures were executed uniformly. Optical density (OD) was measured at 450 nm using a Multiskan GO microplate reader (Thermo Fisher Scientific, USA). Biomarker concentrations were determined by interpolating the OD values against standard curves generated using a 4-parameter logistic (4-PL) curve-fit model.

### 2.4 Clinical laboratory protocols

Routine hematological and biochemical parameters were assessed upon admission using standardized, automated laboratory analyzers. A complete blood count (CBC), including circulating platelet counts, was determined using an automated hematology analyzer Mindray BC-6800Plus (Mindray Bio-Medical Electronics, China).

Systemic biochemical indicators — specifically aspartate aminotransferase (AST), alanine aminotransferase (ALT), total serum protein, urea, and creatinine — were quantified using standard colorimetric and enzymatic assays on the cobas c 303 analytical module of the cobas pure integrated solutions system (Roche Diagnostics, Switzerland).

### 2.5 Statistical analysis

All standard statistical analyses were performed using R software version 4.5.1 (R Foundation for Statistical Computing, Vienna, Austria) within the RStudio integrated development environment. Prior to comparative analyses, missing values within the dataset were addressed using median imputation. Missingness rates prior to imputation were as follows: Creatinine (1.89%), Urea (7.55%), ALT (3.77%), AST (5.66%), Total Bilirubin (13.21%), Direct Bilirubin (43.40%), and Total Protein (26.42%), with 0% missingness for hematological counts. Importantly, this imputation method was applied exclusively to routine clinical biochemical parameters, with no imputation performed on the immunological biomarker data. The normality of data distribution for all continuous variables was assessed using the Shapiro-Wilk test. Continuous variables with a non-normal distribution were expressed as medians and their 25th and 75th percentiles, whereas normally distributed data were presented as means ± standard deviations (SD). Categorical variables were reported as absolute frequencies and percentages. Differences between two independent groups for continuous variables were evaluated using the non-parametric Mann-Whitney U test or the independent samples Student’s t-test. To quantify the magnitude of differences between groups, effect sizes were calculated alongside all continuous comparisons (reported as Rosenthal’s $r=Z/\sqrt{N}$ for Mann-Whitney U tests). Furthermore, to rigorously control for Type I errors across our comprehensive multi-biomarker evaluations, all nominal p-values across all analytical groups (baseline comparative, structural subgroup, and endotype cluster analyses) were subjected to multiple hypothesis testing correction utilizing the Benjamini-Hochberg False Discovery Rate (FDR) procedure. Both nominal and FDR-adjusted p-values, along with their respective effect sizes, are explicitly reported.

Differences between two independent groups for continuous variables were evaluated using the non-parametric Mann-Whitney U test (for non-normally distributed data) or the independent samples Student’s t-test (for normally distributed data). Comparisons of categorical data between groups were performed using Fisher’s exact test. Furthermore, confidence intervals (CI) for percentages were calculated utilizing the exact Fisher’s method. Bivariate correlations between circulating immuno-metabolic biomarkers and routine clinical laboratory parameters were assessed using Spearman’s rank correlation coefficient (*ρ*). A two-tailed p-value of < 0.05 was considered statistically significant for all tests.

Furthermore, to evaluate the impact of structural injury severity on systemic trajectories, a prespecified subgroup comparative analysis was conducted by stratifying the trauma cohort based on the occurrence of major limb amputation.

### 2.6 Unsupervised clustering and machine learning

To identify hidden biological phenotypes (endotypes) within the trauma cohort, unsupervised agglomerative hierarchical clustering was performed. Prior to clustering, the dataset comprising 20 continuous immuno-metabolic and clinical variables was standardized using Z-score scaling to ensure equal weighting across parameters with disparate units of measurement. Clustering was executed using Ward’s minimum variance method (ward.D2) based on Euclidean distances. Prior to final partitioning, the optimal number of clusters (k = 2) was statistically validated utilizing the Average Silhouette Method to ensure maximum inter-cluster distance and intra-cluster cohesion. To visualize the multidimensional separation of these endotypes, Principal Component Analysis (PCA) was conducted, and the resulting clusters were mapped onto a 2D biplot with convex hulls.

Subsequently, a Random Forest classification algorithm was deployed strictly as an exploratory tool (acknowledging the inherent mathematical circularity of evaluating variables that previously defined the clusters) to determine the hierarchy of feature importance and identify the primary biomarkers driving the phenotypic divergence between the clusters. The model was trained using the cluster assignments as the categorical target variable and the continuous biomarkers as predictors. The algorithm was configured to construct 1,000 decision trees (ntree = 1000). The discriminative power of each variable was quantified using the Mean Decrease Gini index, a measure of node impurity that evaluates how much each feature contributes to the homogeneity of the random forest. Higher Mean Decrease Gini values indicate a greater variable contribution to the classification of trauma endotypes. All clustering, dimensionality reduction, and machine learning procedures were executed using the factoextra and randomForest packages in R.

### 2.7 Data and code availability

Data Availability: The clinical and biomarker datasets analyzed during the current study are not publicly available due to institutional ethical restrictions regarding the privacy and confidentiality of military personnel medical data. However, de-identified datasets are available from the corresponding author upon reasonable request and subject to approval by the Institutional Ethics Committee.

Code Availability: The custom R scripts and machine learning codes utilized for the unsupervised clustering and Random Forest analyses in this study are publicly available in the GitHub repository (https://github.com/NefroStat/Trauma-Endotypes-ML) and have been permanently archived on Zenodo at (https://doi.org/10.5281/zenodo.18654533).

## 3 Results

### 3.1 Characterization of the study cohort and baseline biomarker profiling

To evaluate the long-term impact of combat-related trauma on systemic homeostasis, we conducted a comprehensive molecular and clinical analysis of 53 military trauma patients compared with 46 uninjured military controls. By selecting active-duty personnel without injuries as the control group, we aimed to minimize potential confounding factors associated with the combat environment, such as chronic stress and physical exertion. The primary analysis focused on a panel of biomarkers reflecting endothelial integrity, hemodynamic stress, and tissue remodeling, alongside routine clinical laboratory parameters. At baseline, military trauma patients demonstrated significant systemic alterations compared to the control group, specifically characterized by a marked shift in cardiac stress markers and mediators of tissue repair (**Table 1**).

**Table 1:** Baseline characteristics and systemic biomarker profile comparison between military trauma patients and uninjured military controls.

Variable	Military trauma (n = 53) median (25th–75th percentiles)	Uninjured military controls (n = 46) median (25th–75th percentiles)	Nominal P-value	FDR-Adjusted P-value	Effect size (r)
Demographics					
Age (years)	37.0 (32.0–47.0)	48.5 (26.2–55.5)	0.148	0.229	0.163
Sex (Male), n (% [95% CI])	53 (100% [93,2–100%])	46 (100% [92,2–100%])	1.000	N/A	N/A
Biomarker profile					
Soluble ST2 (ng/mL)	13.50 (9.60–19.10)	12.25 (8.40–18.33)	0.324	0.405	0.098
IL1RL1 (ng/mL)	5.07 (0.98–9.96)	6.52 (1.05–13.41)	0.432	0.48	0.078
IL-33 (pg/mL)	25.92 (14.46–111.10)	32.88 (19.87–266.61)	0.160	0.229	0.14
NT-proBNP (pg/mL)	102.49 (80.99–138.33)	147.33 (98.73–207.53)	**< 0.001**	**0.006**	0.341
Annexin V (ng/mL)	13.30 (5.25–31.72)	22.48 (12.06–34.25)	0.068	0.17	0.182
Heparan sulfate (ng/mL)	296.83 (268.67–306.66)	269.28 (261.72–281.76)	**0.002**	**0.008**	0.315
CTGF (pg/mL)	1147.45 (914.72–1576.84)	933.24 (571.29–1267.19)	**0.006**	**0.019**	0.276
TGF-β1 (pg/mL)	2621.95 (2046.05–4022.16)	3207.80 (1277.09–3970.01)	0.925	0.924	**0.01**
Galectin-3 (ng/mL)	5.14 (1.05–10.99)	3.22 (0.48–6.42)	0.105	0.21	0.162

Data are presented as median and their 25th and 75th percentiles. Statistical significance between groups was determined using the Mann-Whitney U test (or Student’s t-test where appropriate). Significant P-values (p < 0.05) are highlighted in bold. Soluble ST2 and IL1RL1 are alternative names representing the same molecular target. CTGF, connective tissue growth factor; IL-33, interleukin-33; IL1RL1, interleukin 1 receptor-like 1; n, number of observations; NT-proBNP, N-terminal pro-B-type natriuretic peptide; ST2, suppression of tumorigenicity 2; TGF-β1, transforming growth factor-beta 1.

### 32 Comparative analysis of baseline biomarker profiles

**Figure 1:**
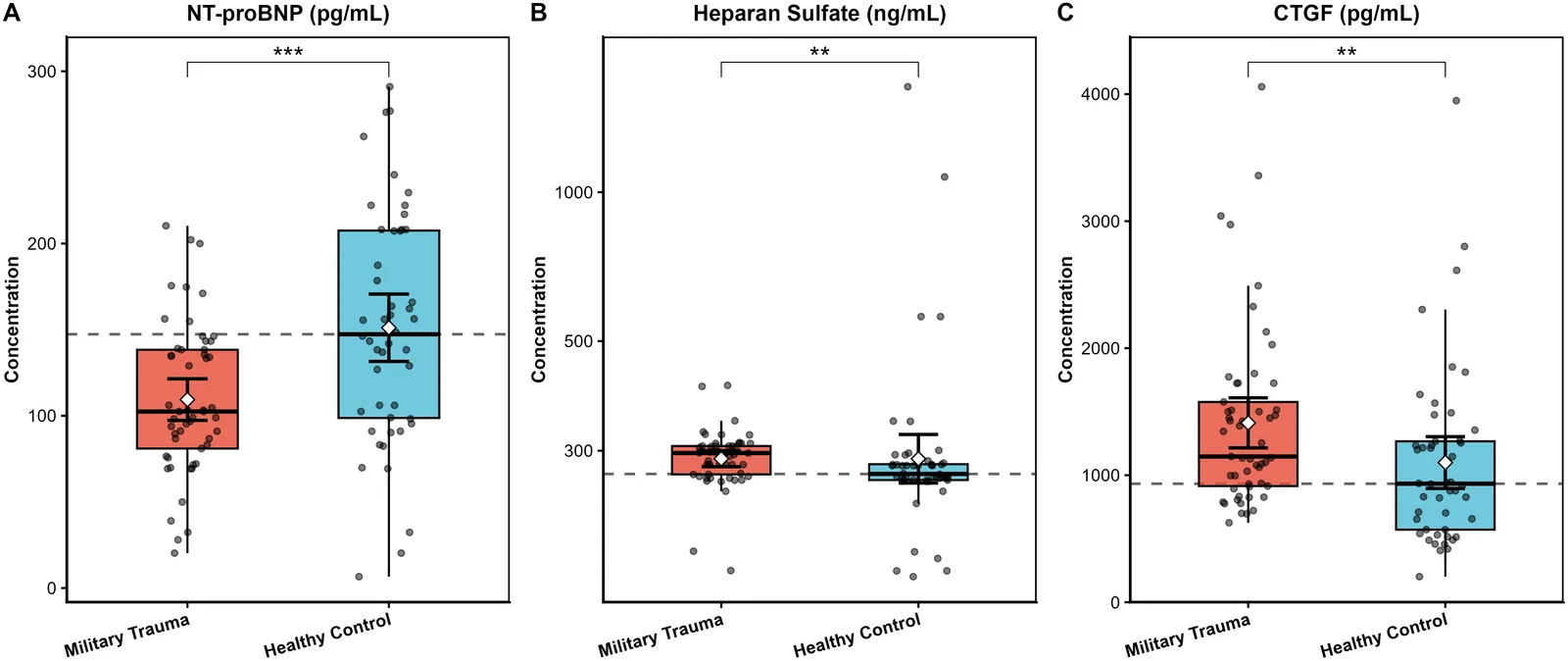
Systemic concentrations of NT-proBNP, heparan sulfate, and CTGF are significantly altered in military trauma patients. Systemic levels of **(A)** NT-proBNP, **(B)** heparan sulfate, and **(C)** CTGF were compared between military trauma patients (n = 53) and uninjured military controls (n = 46). Boxplots display the median (solid horizontal line) and their 25th and 75th percentiles. Whiskers extend to the most extreme data points within 1.5 × IQR. Individual raw data points (jitter) are overlaid to illustrate the underlying data distribution. The white diamond symbol represents the sample mean, with error bars indicating the 95% confidence interval (CI). The horizontal dashed line across each panel indicates the median value of the uninjured control group as a reference baseline. Note that the y-axis in panel **(B)** is presented on a logarithmic (log10) scale to accommodate high data dispersion. Statistical significance was evaluated using the Mann-Whitney U test (for heparan sulfate and CTGF) or Student’s t-test (for NT-proBNP); **P < 0.01, ***P < 0.001

Comparative analysis revealed significant systemic divergences between military trauma patients and the control group. Indicators of hemodynamic stress, specifically N-terminal pro-B-type natriuretic peptide (NT-proBNP) levels, were significantly lower in the trauma cohort compared to uninjured military controls (*P* < 0.001; **Figure 1A**). In contrast, markers of endothelial injury and fibrosis demonstrated an opposite trend. Heparan sulfate levels were markedly elevated in trauma patients (*P* = 0.002; **Figure 1B**), as was the concentration of CTGF (*P* = 0.006; **Figure 1C**).

No statistically significant differences were observed between the groups for other evaluated biomarkers, including soluble ST2, IL-33, annexin V, and TGF-β1 at this stage of the analysis. Overall, despite inter-individual overlap, the trauma cohort presented with elevated markers of glycocalyx degradation and extracellular matrix remodeling, alongside lower systemic NT-proBNP concentrations. However, these lower NT-proBNP levels must be interpreted with caution, as they are likely attributable to the significantly lower median age of the trauma cohort compared to the control group.

### 3.3 Lack of temporal decay in the trauma-induced biomarker profile

To determine whether the substantial biomarker alterations observed in the military trauma cohort were acute transient responses or persistent systemic shifts, we evaluated the temporal dynamics by correlating biomarker concentrations with the time elapsed since the initial injury (days since injury).

**Figure 2:**
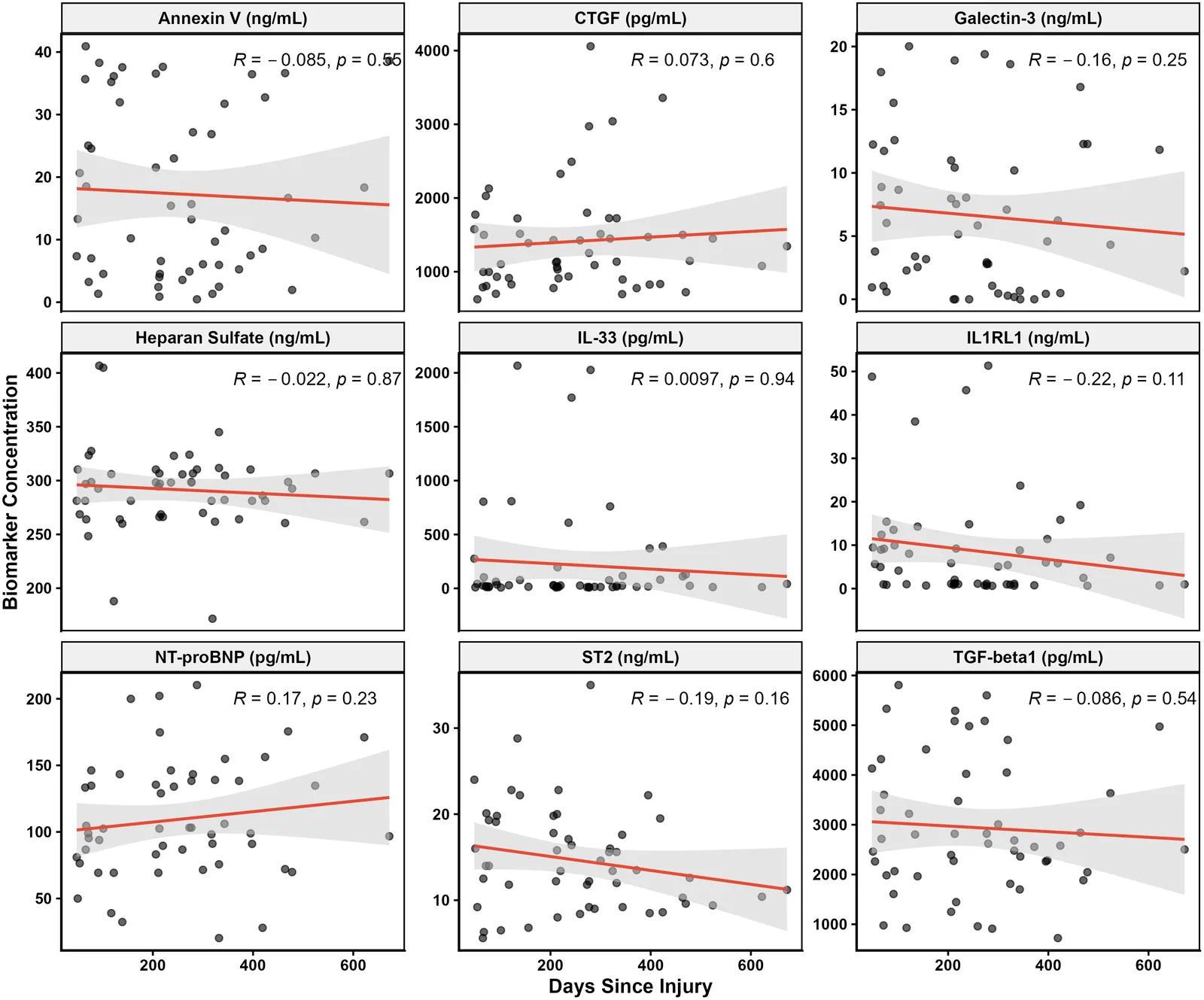
Temporal dynamics of systemic biomarkers following military trauma. Spearman rank correlation analysis evaluating the relationship between biomarker concentrations and the time elapsed since the initial injury (days) in the military trauma cohort (n = 53). Scatter plots display individual patient data points with a linear regression trendline (red) and the corresponding 95% confidence interval (gray shaded area). Spearman’s correlation coefficients (r) and statistical significance (P values) are annotated within each panel. No significant temporal correlations were observed across the biomarker panel, indicating a persistent and stable state of systemic dysregulation independent of recovery time. Soluble ST2 and IL1RL1 are alternative names representing the same molecular target.

Remarkably, Spearman rank correlation analysis revealed no significant temporal dependence for any of the evaluated biomarkers. Specifically, markers of endothelial dysfunction and extracellular matrix remodeling remained stably altered regardless of the recovery duration, including heparan sulfate (*r* = -0.022, *P* = 0.873), CTGF (*r* = 0.073, *P* = 0.602), and galectin-3 (*r* = -0.161, *P* = 0.248). Similarly, markers associated with hemodynamic stress and immune activation, such as NT-proBNP (*r* = 0.166, *P* = 0.234) and soluble ST2 (*r* = -0.194, *P* = 0.165), showed no significant trend towards normalization over time. Other systemic mediators, including interleukin 1 receptor-like 1 (IL1RL1; *r* = -0.221, *P* = 0.113), annexin V (*r* = -0.085, *P* = 0.546), TGF-β1 (*r* = -0.086, *P* = 0.543), and IL-33 (*r* = 0.010, *P* = 0.945), also lacked significant temporal correlation (**Figure 2**).

Overall, the lack of significant temporal correlation indicates that these systemic biomarker profiles remain stable regardless of the recovery duration, demonstrating that the observed molecular variations are not strictly driven by the simple passage of time since the initial injury.

### 3.4 Impact of severe structural injury on molecular and clinical trajectories

Given the sustained elevation of the systemic immune-endothelial stress response across the rehabilitation timeline in our cohort, we next sought to determine whether the physical extent and severity of the initial trauma correlate with specific molecular and clinical outcomes. To address this, we stratified the trauma cohort based on the occurrence of traumatic limb amputation (amputation: yes, n = 21; amputation: no, n = 32) and analyzed their comparative biomarker profiles and routine laboratory parameters (**Table 2**).

**Table 2:** Impact of traumatic amputation on systemic biomarkers, clinical laboratory parameters, and infection rates within the military trauma cohort.

Variable	Amputation: No (n = 32) median (25th–75th percentiles)	Amputation: Yes (n = 21) median (25th–75th percentiles)	Nominal P-value	FDR-Adjusted P-value	Effect size (r)
Demographics					
Age (years)	39.5 (32.8–49.3)	37.0 (29.0–40.0)	0.045	0.534	0.21
Sex (Male), n (%; 95% CI)	32 (100%; 95% CI [89,1–100%])	21 (100%; 95% CI [83,9–100%])	1	N/A	N/A
Biomarkers					
Soluble ST2 (ng/mL) n=53	15.70 (10.80–19.80)	12.50 (9.20–15.60)	0.114	0.534	0.219
IL1RL1 (ng/mL) n=53	5.85 (1.00–11.66)	4.97 (0.97–8.95)	0.617	0.928	0.07
IL-33 (pg/mL) n=53	25.88 (15.20–108.99)	26.43 (12.64–111.10)	0.656	0.928	0.062
NT-proBNP (pg/mL) n=53	102.85 (82.60–138.51)	98.91 (76.47–138.33)	0.915	0.928	0.03
Annexin V (ng/mL) n=53	16.18 (6.42–32.15)	10.21 (4.92–26.87)	0.495	0.928	0.095
Heparan Sulfate (ng/mL) n=53	298.19 (278.04–306.66)	292.49 (268.67–306.66)	0.592	0.928	0.075
CTGF (pg/mL) n=53	1142.34 (913.33–1529.88)	1388.71 (996.45–1726.23)	0.723	0.928	0.05
TGF-β1 (pg/mL) n=53	2337.80 (1783.98–2920.18)	3294.77 (2554.83–4704.17)	0.004	0.084	0.395
Galectin-3 (ng/mL) n=53	5.21 (1.93–10.56)	5.14 (0.46–11.83)	0.834	0.928	0.03
Clinical laboratory					
Creatinine (µmol/L) n=52	77.5 (67.9–87.4), n=31	72.3 (61.4–101.0), n=21	0.676	0.928	0.059
Urea (mmol/L) n=49	5.18 (4.62–6.31), n=31	5.45 (5.10–5.89), n=18	0.771	0.928	0.031
ALT (U/L) n=51	27.5 (21.0–50.3), n=31	22.7 (13.4–37.7), n=20	0.424	0.928	0.109
AST (U/L) n=52	23.1 (18.8–39.3), n=30	23.1 (19.8–25.5), n=20	0.49	0.928	0.108
Total bilirubin (µmol/L) n=46	8.99 (6.25–12.25), n=27	8.50 (5.50–9.60), n=19	0.248	0.928	0.153
Direct bilirubin (µmol/L) n=29	4.00 (3.85–4.54), n=17	4.00 (3.40–4.00), n=12	0.415	0.928	0.158
Total Protein (g/L) n=39	68.6 (63.2–72.8), n=23	67.3 (64.2–72.3), n=16	0.991	0.928	0.018
Hemoglobin (g/L) n=53	139.5 (122.3–146.5), n=32	115.0 (97.0–138.0), n=21	0.133	0.534	0.207
WBC (×10^9^/L) n=53	7.03 (5.67–9.54), n=32	7.50 (5.66–8.34), n=21	0.928	0.928	0.014
Platelets (×10^9^/L) n=53	234.0 (205.5–329.0), n=32	354.0 (238.0–468.0), n=21	0.02	0.204	0.32
Complications					
Persistent localized wound/stump infections (n, %)	2 (6.2%; 95% CI [0.8–20.8%])	9 (42.9%; 95% CI [21.8–66.0%])	0.004	N/A	N/A

Continuous data are presented as median and their 25th and 75th percentiles. Categorical data are presented as counts and percentages with exact 95% confidence intervals (CI). Statistical comparisons were performed using the Mann-Whitney U test, Student’s t-test, or Fisher’s exact test, as appropriate based on data distribution. Significant differences (P < 0.05) are highlighted in bold. The exact number of available true observations (n) prior to median imputation is explicitly stated directly within the table for all routine clinical biochemical parameters, detailing the distribution between the respective subgroups. Soluble ST2 and IL1RL1 are alternative names representing the same molecular target. ALT, alanine aminotransferase; AST, aspartate aminotransferase; CI, confidence interval; CTGF, connective tissue growth factor; IL-33, interleukin-33; IL1RL1, interleukin 1 receptor-like 1; n, number of observations; NT-proBNP, N-terminal pro-B-type natriuretic peptide; ST2, suppression of tumorigenicity 2; TGF-β1, transforming growth factor-beta 1; WBC, white blood cells.

**Figure 3:**
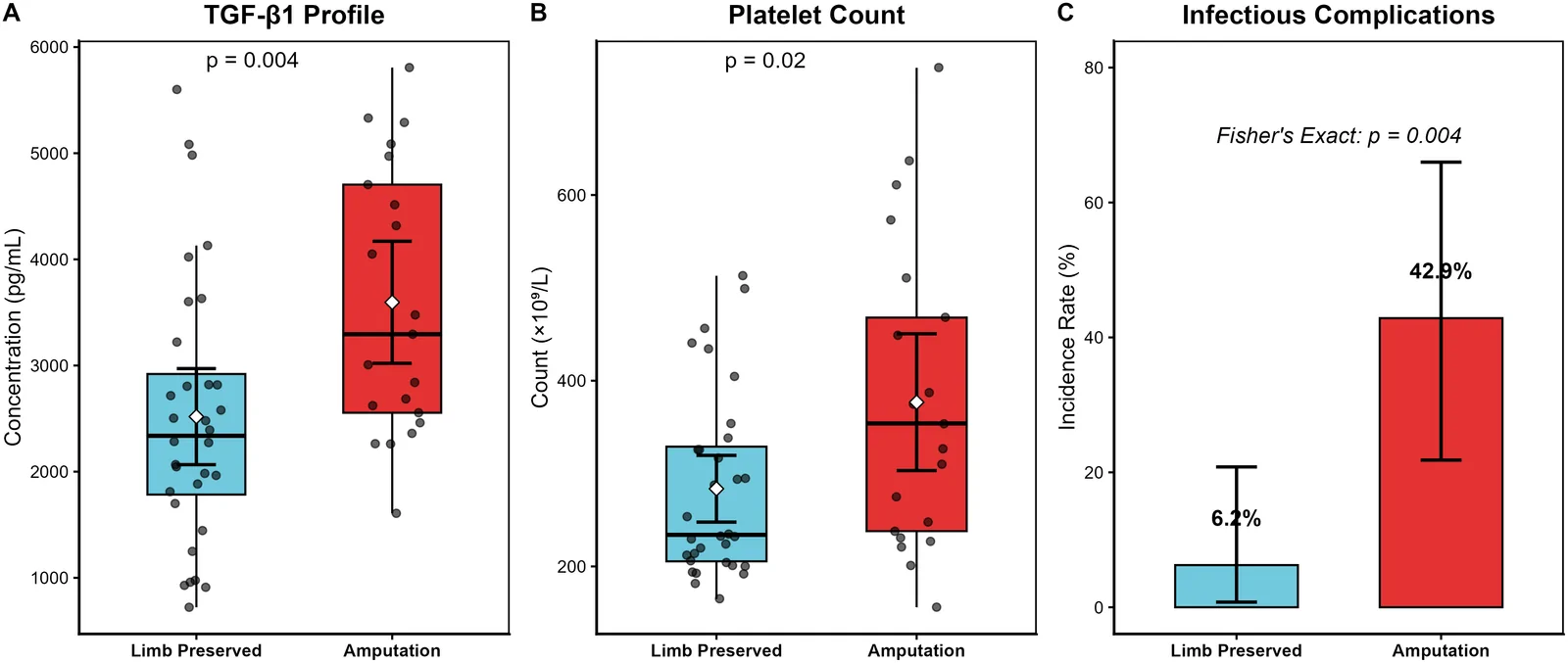
Traumatic limb amputation is associated with a distinct pro-fibrotic, pro-coagulant, and infectious-vulnerable clinical phenotype. The military trauma cohort (n = 53) was stratified by the occurrence of major limb amputation (amputation: yes, n = 21; amputation: no, n = 32). **(A)** Circulating levels of the pro-fibrotic mediator TGF-β1 and **(B)** systemic platelet counts were significantly elevated in the amputated subgroup. For continuous variables, boxplots represent the median (25th and 75th percentiles), with overlaid jitter points showing individual patient distribution. White diamonds denote the sample mean ± 95% confidence interval (CI). **(C)** The incidence of secondary infectious complications was drastically higher in patients with amputations. Bar heights represent the incidence rate (%), and error bars indicate the exact binomial 95% confidence intervals (Clopper-Pearson method). Statistical significance was determined using the Student’s t-test or Mann-Whitney U test for continuous variables, and Fisher’s exact test for categorical incidence data. Unadjusted nominal p-values are reported directly on panels A–C; after FDR correction across the continuous marker panel, TGF-β1 and platelet counts represent statistical trends (FDR p = 0.084 and FDR p = 0.204, respectively). Abbreviations: TGF-β1, transforming growth factor-beta 1.

Subgroup analysis revealed higher levels of the pro-fibrotic mediator TGF-β1 (nominal P = 0.004, effect size r = 0.395) and elevated platelet counts (nominal P = 0.020, r = 0.320) in patients with traumatic amputations compared to non-amputated patients. However, following FDR correction across the marker panel, these continuous parameters transitioned to statistical trends (FDR-adjusted P = 0.084 and P = 0.204, respectively), reflecting the constrained statistical power inherent to partitioning a small cohort (n = 21 vs. 32). In contrast, the most critical clinical divergence between these subgroups was the significantly higher incidence of persistent localized infections in the amputated cohort (Fisher’s exact P = 0.004). Given the strong effect size of TGF-β1 and considering the well-established role of infection in driving reactive thrombocytosis and extracellular matrix remodeling, it is highly probable that the ongoing infectious burden in large residual limb wounds acts as a primary confounding driver for these observed pro-fibrotic and pro-coagulant trends. Rather than structural tissue loss alone initiating an isolated response, the heightened infectious vulnerability of residual limbs appears to substantially modulate the systemic remodeling trajectory. (**Figure 3**).

### 3.5 Unsupervised clustering identifies a distinct hyper-inflammatory alarmin-driven endotype

**Figure 4:**
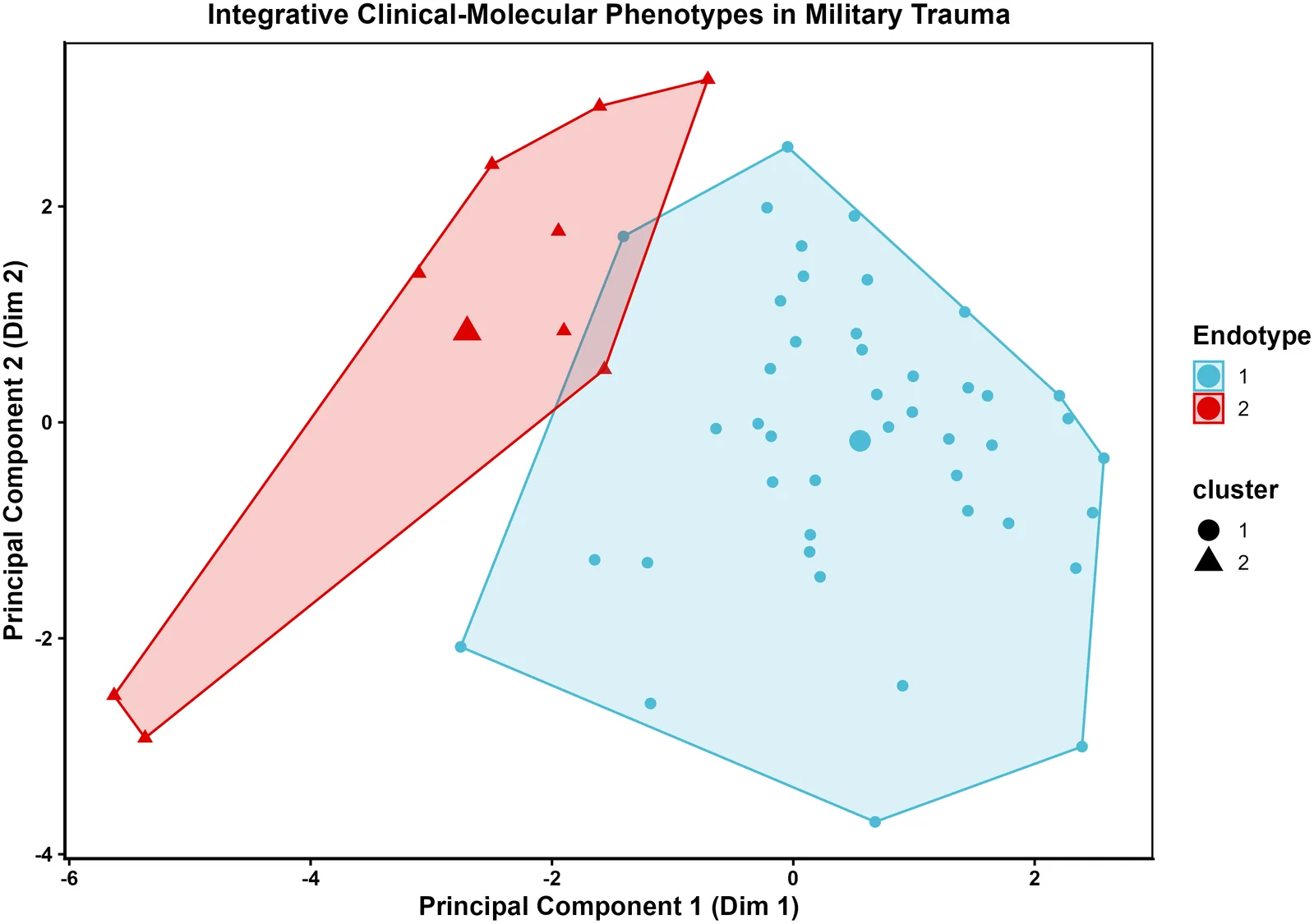
Principal component analysis (PCA) reveals two distinct integrative clinical-molecular endotypes in military trauma. Unsupervised agglomerative hierarchical clustering (Ward’s method) was performed on a scaled dataset comprising 20 continuous variables (systemic biomarkers and routine clinical laboratory parameters) exclusively within the military trauma cohort (n = 53). The resulting two-dimensional PCA plot visualizes the spatial distribution of patients into two distinct phenotypic clusters (Endotype 1 and Endotype 2). Individual data points represent unique patients, while the shaded convex hulls delineate the multidimensional boundaries of each endotype. The axes (Dim 1 and Dim 2) represent the first and second principal components, respectively, capturing the maximum variance within the integrated dataset. The larger, solid highlighted points (circle and triangle) represent the geometric centroids of their respective phenotypic clusters.

While stratifying by structural injury (amputation) revealed a specific pro-fibrotic trajectory, combat trauma is associated with a heterogeneous systemic response that may not be fully captured by anatomical classifications alone. To determine whether broader, underlying molecular phenotypes exist independent of predefined clinical labels, we employed an unsupervised agglomerative hierarchical clustering approach using the integrated dataset of 20 continuous variables (biomarkers and clinical laboratory parameters). This analysis separated the trauma cohort into two distinct clinical-molecular phenotypes, designated as Endotype 1 (n = 44) and Endotype 2 (n = 9) (**Figure 4**).

Comparative analysis of the two identified endotypes revealed substantial molecular and clinical divergences (**Table 3**). Endotype 2 (n = 9) was characterized by an extreme elevation of IL-33, a potent tissue-derived alarmin (P = 0.001). This massive IL-33 release suggests widespread cellular necrosis and severe tissue disruption. To ensure that this extreme IL-33 elevation was not solely driven by a single statistical outlier, a prespecified sensitivity analysis was performed excluding the highest IL-33 value (2066.60 pg/mL) within Endotype 2. This analysis confirmed that circulating IL-33 levels remained robustly and significantly elevated in Endotype 2 compared to Endotype 1 (nominal p = 0.003), validating the stability of this alarmin-driven systemic signature.

**Table 3:** Clinical and molecular characterization of the two identified trauma endotypes.

Variable	Endotype 1 (n = 44) median (25th–75th percentiles)	Endotype 2 (n = 9) median (25th–75th percentiles)	Nominal P-value	FDR-Adjusted P-value	Effect size (r)
Demographics					
Age (years)	36.5 (31.8–47.0)	41.0 (38.0–45.0)	0.184	0.23	0.184
Sex (Male), n (% [95% CI])	44 (100% [92,0–100%])	9 (100% [66,4–100%])	1.000	N/A	N/A
Biomarkers					
Soluble ST2 (ng/mL) n=53	13.45 (9.20–17.65)	16.00 (12.50–22.80)	0.118	0.165	0.216
IL1RL1 (ng/mL) n=53	3.30 (0.98–9.28)	8.95 (5.41–19.21)	0.121	0.165	0.215
IL-33 (pg/mL) n=53	22.67 (12.56–75.97)	760.49 (61.51–808.57)	0.001	0.023	0.449
NT-proBNP (pg/mL) n=53	103.21 (88.88–138.51)	72.06 (69.31–91.18)	0.049	0.123	0.272
Annexin V (ng/mL) n=53	12.35 (5.78–24.69)	31.95 (1.96–36.13)	0.374	0.44	0.124
Heparan Sulfate (ng/mL) n=53	298.19 (278.35–310.27)	292.48 (260.55–296.83)	0.086	0.16	0.238
CTGF (pg/mL) n=53	1195.54 (925.31–1613.87)	1147.45 (914.72–1500.83)	0.878	0.915	0.023
TGF-β1 (pg/mL) n=53	2567.26 (2046.06–4028.99)	2804.08 (2046.05–3220.96)	0.915	0.915	0.016
Galectin-3 (ng/mL) n=53	4.86 (0.88–9.21)	12.28 (2.80–16.79)	0.123	0.165	0.213
Clinical laboratory					
Creatinine (µmol/L) n=52	78.1 (67.9–97.9), n=43	61.0 (52.6–85.3), n=9	0.088	0.16	0.236
Urea (mmol/L) n=49	5.45 (4.79–6.13), n=40	5.80 (4.19–7.00), n=9	0.713	0.793	0.052
ALT (U/L) n=51	24.3 (16.9–36.5), n=42	52.5 (37.9–104.0), n=9	0.009	0.035	0.361
AST (U/L) n=52	21.9 (17.9–27.1), n=42	41.8 (25.5–81.0), n=8	0.005	0.024	0.391
Total bilirubin (µmol/L) n=46	8.98 (6.75–13.47), n=37	5.90 (4.30–9.30), n=9	0.107	0.165	0.223
Direct bilirubin (µmol/L) n=29	4.00 (4.00–4.54), n=24	3.81 (2.55–4.00), n=6	0.086	0.16	0.238
Total Protein (g/L) n=39	68.2 (67.4–72.2), n=31	60.1 (58.1–66.4), n=8	0.005	0.024	0.388
Hemoglobin (g/L) n=53	138.5 (118.8–148.5), n=44	99.0 (86.0–109.0), n=9	0.005	0.024	0.391
WBC (×10^9^/L) n=53	6.61 (5.49–8.17), n=44	8.72 (7.86–9.90), n=9	0.012	0.036	0.345
Platelets (×10^9^/L) n=53	251.0 (210.5–342.0), n=44	434.0 (375.0–513.0), n=9	0.011	0.036	0.351
Clinical outcomes					
Amputation (n, % [95% CI])	16 (36.4% [22.4–52.2%])	5 (55.6% [21.2–86.3%])	0.456	N/A	N/A
Persistent localized wound/stump infections (n, % [95% CI])	8 (18.2% [8.2–32.7%])	3 (33.3% [7.5–70.1%])	0.372	N/A	N/A

Continuous data are presented as median and their 25th and 75th percentiles. Categorical data are presented as counts and percentages with exact binomial 95% confidence intervals (CI). Statistical significance (P < 0.05) is highlighted in bold and was assessed using the Mann-Whitney U test, Student’s t-test, or Fisher’s exact test. The exact number of available true observations (n) prior to median imputation is explicitly stated directly within the table for all routine clinical biochemical parameters, detailing the distribution between Endotype 1 and Endotype 2. Soluble ST2 and IL1RL1 are alternative names representing the same molecular target. ALT, alanine aminotransferase; AST, aspartate aminotransferase; CI, confidence interval; CTGF, connective tissue growth factor; IL-33, interleukin-33; IL1RL1, interleukin 1 receptor-like 1; n, number of observations; NT-proBNP, N-terminal pro-B-type natriuretic peptide; ST2, suppression of tumorigenicity 2; TGF-β1, transforming growth factor-beta 1; WBC, white blood cells.

Corroborating this molecular signature, patients in Endotype 2 exhibited a systemic profile consistent with critical illness and severe systemic stress. This included significantly higher levels of hepatic injury markers (AST: P = 0.005; ALT: P = 0.009), pronounced anemia (P = 0.005), and hypoproteinemia (P = 0.005), consistent with a pronounced systemic inflammatory state and secondary organ strain. While these findings might suggest catabolic shifts, they strongly align with the higher infection frequency in this subgroup and potential medication-related hepatic strain.

Although clinical complications such as amputation and subsequent infections were observed at higher frequencies in Endotype 2, these trends did not reach statistical significance, likely due to the limited sample size of this extreme phenotype subgroup. Collectively, these findings demonstrate that unsupervised clustering successfully isolated a **“**hyper-inflammatory, alarmin-driven**”** phenotype associated with massive tissue injury and severe systemic destabilization following combat trauma.

### 3.6 Identification of key phenotypic features via random forest analysis

**Figure 5:**
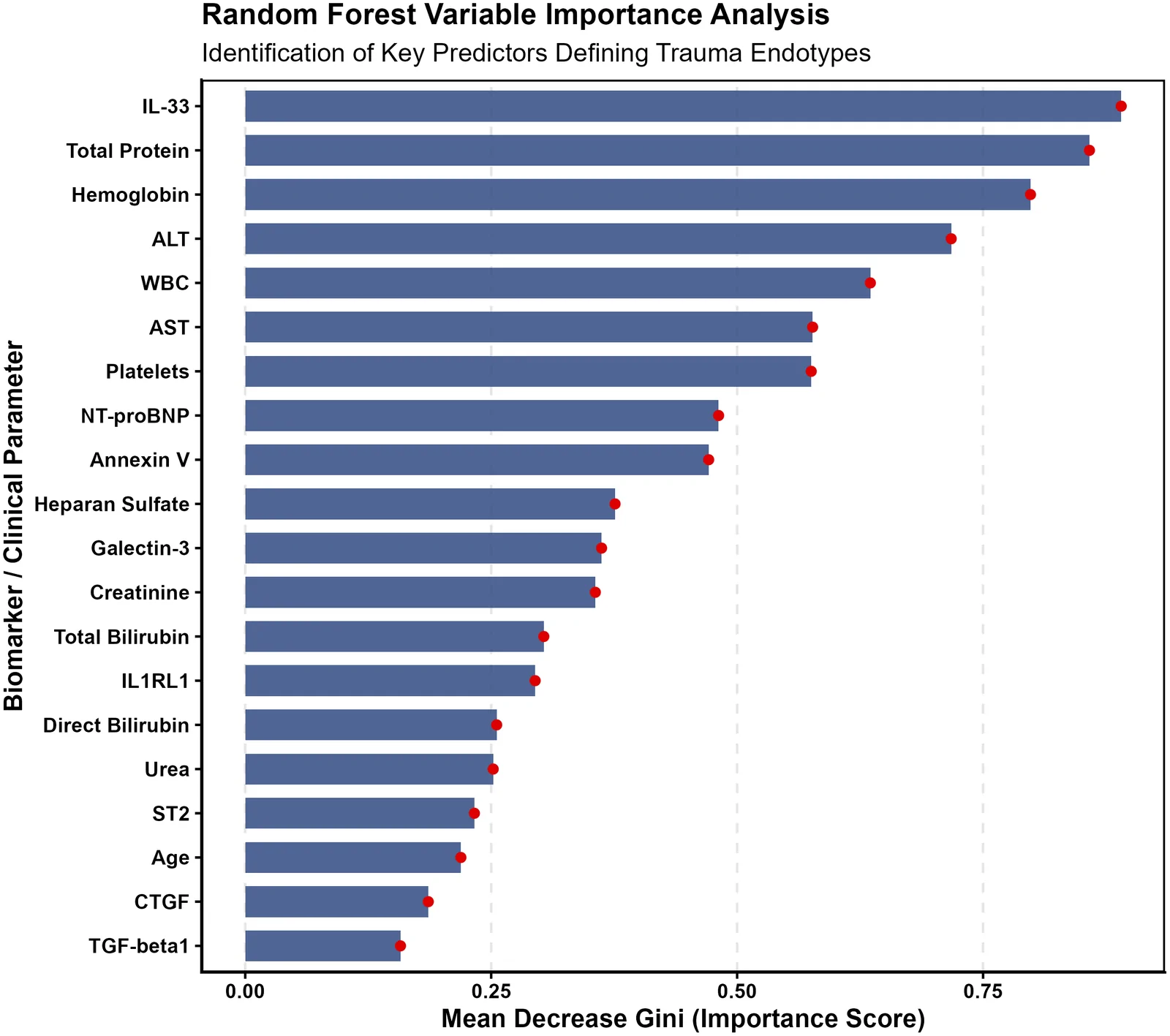
Machine learning-based identification of key biological drivers of military trauma endotypes. A class-balanced Random Forest classification model (1,000 trees) ranked the predictive importance of 20 integrated variables in defining the identified trauma molecular endotypes. Variable importance was quantified using the mean decrease Gini coefficient, where higher scores indicate a greater contribution to the classification accuracy. The analysis identifies the tissue-derived alarmin IL-33, along with markers of metabolic and hematological stress (total protein, hemoglobin, ALT, WBC, and AST), as the primary predictors of phenotypic divergence in military trauma patients. ALT, alanine aminotransferase; AST, aspartate aminotransferase; CTGF, connective tissue growth factor; IL-33, interleukin-33; NT-proBNP, N-terminal pro-B-type natriuretic peptide; ST2, suppression of tumorigenicity 2; TGF-β1, transforming growth factor-beta 1; WBC, white blood cells.

To ensure the internal validity of the machine learning approach and mitigate the risk of overfitting due to the pronounced class imbalance (Endotype 1, n = 44 vs. Endotype 2, n = 9), we utilized a class-balanced Random Forest model with downsampling. The model achieved a robust overall out-of-bag (OOB) error rate of 15.09%. While the algorithm demonstrated high accuracy in identifying the predominant phenotype (Class 1 error rate: 6.8%), the discriminative capacity for the minority severe phenotype was more limited (Class 2 error rate: 55.6%), reflecting the inherent mathematical challenges of modeling small, heterogeneous biological sub-cohorts. Despite this limitation in strict boundary resolution, the model’s overall internal validity was deemed sufficient for its primary exploratory objective: extracting and ranking the hierarchical importance of the underlying biological features (**Figure 5**).

The tissue-damage alarmin IL-33 emerged as the most critical variable (mean decrease Gini ~ 0.9), highlighting its prominence in differentiating the hyper-inflammatory trauma phenotype. This was followed by a cluster of routine clinical markers reflecting an ongoing inflammatory response, infectious burden, and associated supportive pharmacotherapy.

Notably, while biomarkers such as platelets, NT-proBNP, and annexin V contributed moderately to the model’s predictive power, the soluble IL-33 receptor (ST2) ranked near the bottom of the importance hierarchy. This observation directly supports our hypothesis that the rehabilitation systemic response is associated with the continuous release of the IL-33 ligand from damaged tissues, rather than by systemic modulation of its receptor. Furthermore, established fibrosis markers (CTGF, TGF-β1) ranked lowest in the feature hierarchy. These findings suggest that the post-traumatic divergence in military trauma phenotypes is primarily characterized by an “alarmin-metabolic-hematological” axis, rather than established cardiovascular or late-stage fibrotic pathways.

## 4 Discussion

In this study, we characterized the systemic molecular landscape of military trauma during the rehabilitation phase of recovery, utilizing a cohort of uninjured active-duty personnel as a baseline control. Our findings support the hypothesis that severe blast and penetrating injuries are associated with a protracted state of systemic dysregulation that extends beyond the initial acute response (16). While traditional clinical assessments highlight a pro-fibrotic and endothelial-damaging response associated with massive structural tissue loss, we observed substantial hidden heterogeneity among survivors (17). By applying unsupervised clustering and Random Forest classification to our multidimensional data, our analysis suggests that the formation of clinical endotypes is not solely explained by anatomical injury severity (18). Instead, phenotypic divergence in the rehabilitation phase appears to be primarily associated with a distinct immuno-metabolic axis. This axis is defined by the elevation of the tissue-damage mediator IL-33 (19), alongside markers of systemic metabolic and hepatic strain (AST, ALT, and total protein) (20), rather than by classical markers of late-stage fibrosis alone.

A critical aspect of the post-traumatic response observed in our cohort is the sustained degradation of the vascular endothelial glycocalyx, evidenced by elevated circulating heparan sulfate. While acute glycocalyx shedding is a well-documented marker of initial shock, our findings suggest that severe structural injury is accompanied by an endotheliopathy that persists well into the rehabilitation phase (21). Importantly, the presence of persistent localized wound or stump infections in a subset of trauma patients (11 out of 53) likely acts as a potent secondary driver for both glycocalyx shedding (elevated heparan sulfate) and pro-fibrotic activation (CTGF), underscoring the synergistic impact of severe tissue trauma and chronic localized infection. Furthermore, stratification of our cohort revealed that patients who suffered massive structural tissue loss, specifically traumatic limb amputations, exhibited a distinct pro-fibrotic and pro-thrombotic signature. This subgroup demonstrated significantly elevated levels of TGF-β1, a cytokine known to mediate complex tissue remodeling and dysregulated wound healing in severe extremity trauma (22, 23). The concurrent presence of elevated TGF-β1 and reactive thrombocytosis within the amputated subgroup must be interpreted in the context of their significantly higher infectious burden (42.9%). Rather than reflecting an isolated response to structural tissue loss (24, 25), this altered hematological and pro-fibrotic profile is likely driven by the chronic localized infections of the residual limbs. Such ongoing infectious burden acts as a substantial persistent stimulus for systemic inflammation and maladaptive tissue remodeling, effectively maintaining the patient in a prolonged state of physiological vulnerability (26).

To explore the biological variability within our trauma cohort, we applied unsupervised clustering and Random Forest classification (27). This analysis identified the tissue-damage marker IL-33 as the strongest predictor of patient endotypes, outperforming classical pro-fibrotic markers such as CTGF. Notably, while circulating IL-33 levels varied significantly across these groups, the concentration of its soluble receptor, ST2, remained stable (28, 29). This suggests that the systemic response in the rehabilitation phase is associated with the continuous release of IL-33 from damaged tissues, rather than systemic changes in receptor expression. Furthermore, the Random Forest model highlighted hepatic and metabolic markers — specifically AST, ALT, and total protein — as key features defining these endotypes (30). Together, these findings point to a distinct “immuno-metabolic” response. The strong link between ongoing tissue damage (IL-33) and hepatic strain suggests that patients in the high-risk endotype are likely experiencing substantial systemic and metabolic exhaustion (31). Importantly, the higher incidence of localized secondary infections observed in extreme phenotype subgroups (such as Endotype 2 and amputees) raises the possibility that a chronic infectious burden may synergistically amplify this alarmin-driven response. Differentiating the primary trauma-induced molecular signature from secondary infection-driven inflammation remains a complex challenge that requires further investigation.

A major strength of our study is the utilization of an uninjured active-duty military cohort as a baseline control. This approach allowed us to rigorously isolate the molecular signature of severe structural trauma from the baseline physiological stress and physical exertion inherent to military deployment (32). Clinically, our findings underscore that relying solely on the anatomical classification of wounds — such as the mechanism of injury or the presence of a limb amputation — is insufficient to predict a patient’s systemic recovery trajectory (33). The substantial biological heterogeneity observed among patients with comparable structural injuries highlights the critical need for molecular phenotyping (34). Integrating immuno-metabolic biomarker profiling with machine learning algorithms could facilitate the early identification of trauma survivors at high risk for systemic exhaustion, thereby paving the way for targeted, precision-medicine interventions (35).

When interpreting the laboratory features defining Endotype 2, pragmatic clinical explanations must be considered alongside biological pathways. While extreme IL-33 elevation represents a prominent alarmin-driven feature, the accompanying laboratory abnormalities — specifically anemia, leukocytosis, thrombocytosis, and hypoproteinemia — are characteristic of persistent, low-grade systemic inflammation secondary to chronic localized infections. Indeed, Endotype 2 demonstrated an 80% higher prevalence of persistent wound/stump infections compared to Endotype 1. Furthermore, the mild transaminase elevations (ALT and AST) observed in Endotype 2 likely reflect subclinical hepatic stress, which can be partially driven by prolonged supportive pharmacotherapy, including prolonged antimicrobial regimes and analgesics. These observations align with long-term follow-up studies in major trauma survivors, which describe persistent immune dysregulation and ongoing inflammatory-metabolic strain — such as the Persistent Inflammation, Immunosuppression, and Catabolism Syndrome (PICS) — rather than acute shock-related phenomena like capillary leak during late rehabilitation (37, 38).

Despite these insights, several limitations must be acknowledged. First, this was a single-center, cross-sectional study conducted during the rehabilitation phase, which limits our ability to track longitudinal changes from the point of injury. Consequently, the absence of temporal biomarker decay observed in our cohort may be subject to survivorship bias, reflecting the persistence of severe cases in the rehabilitation facility rather than intra-individual biomarker stability. Second, while granular details regarding the precise technical physics of the primary injury and exact timing relative to the final surgical revision were partially restricted due to multi-echelon evacuation, all patients in this cohort shared a uniform primary injury mechanism (mine-blast trauma), which significantly minimizes confounding bias related to varying trauma modalities. Third, patients received standard-of-care supportive medications during rehabilitation, including analgesics, antidepressants, and systemic or local antimicrobial therapy for persistent wound/stump infections; while reflecting real-world clinical management, these treatments could exert immunomodulatory effects. Furthermore, although the control group was screened via outpatient records for active chronic conditions, potential unmeasured subclinical confounders and age disparities between groups (which may partially explain the relatively lower baseline NT-proBNP levels in the younger trauma cohort) cannot be completely ruled out. Finally, the relatively small sample size precluded the use of an independent holdout set for cross-validation. Additionally, the limited sample size within the extreme phenotype cluster (n = 9) mathematically precluded robust multivariable covariate adjustment to statistically isolate the independent effect of infection status from the primary trauma signature. Consequently, we explicitly acknowledge the inherent mathematical circularity in utilizing a Random Forest model to rank the importance of the exact same features used to derive the hierarchical clusters. Thus, the Random Forest findings must be interpreted strictly as an exploratory descriptive ranking of the endotypes rather than an independently validated predictive clinical model. Future research in larger, multicenter cohorts is necessary to independently validate these endotypes (36). Future research should focus on longitudinal monitoring to determine whether these specific molecular signatures strictly correlate with long-term clinical outcomes, such as chronic pain or persistent organ dysfunction.

In conclusion, our findings suggest that severe combat-related trauma is associated with a stable and heterogeneous molecular imprint that persists into the rehabilitation phase. By employing unsupervised clustering and machine learning algorithms, we identified that this phenotypic divergence appears to be closely associated with a distinct immuno-metabolic axis — characterized by elevated IL-33 and concurrent systemic metabolic strain — rather than by structural injury patterns or classical fibrotic markers alone. These results support the potential clinical utility of transitioning from purely anatomical classifications of combat wounds toward comprehensive molecular endotyping. Identifying high-risk molecular profiles during early rehabilitation may offer a vital window of opportunity for personalized clinical monitoring. Ultimately, integrating these immuno-metabolic signatures into military medicine could provide a foundation for mitigating systemic exhaustion and improving long-term recovery trajectories in trauma survivors.

## Data Availability

The original contributions presented in the study are included in the article/**Supplementary Material**. Further inquiries can be directed to the corresponding author/s.
